# Working posture and its predictors in hospital operating room nurses

**DOI:** 10.15171/hpp.2016.03

**Published:** 2016-03-31

**Authors:** Farahnaz Abdollahzade, Fariba Mohammadi, Iman Dianat, Elnaz Asghari, Mohammad Asghari-Jafarabadi, Zahra Sokhanvar

**Affiliations:** ^1^Faculty of Nursing and Midwifery, Tabriz University of Medical Sciences, Tabriz, Iran; ^2^Department of Occupational Health, Tabriz University of Medical Sciences, Tabriz, Iran; ^3^Human Factors Research Group, University of Nottingham, Nottingham, UK; ^4^Road Traffic Injury Research Center, Tabriz University of Medical Sciences, Tabriz, Iran

**Keywords:** REBA, Postural, Nursing, Healthcare, Iran

## Abstract

**Background: ** This study was conducted to evaluate working posture of operating room nurses and its relationship with demographic and job details of this group.

**Methods: ** This cross-sectional study was conducted among 147 operating room nurses in Tabriz, Iran using a questionnaire and the Rapid Entire Body Assessment (REBA) checklist. The data were analyzed with SPSS.16 using t test, Pearson correlation coefficient and analysis of variance (ANOVA) tests for univariate analysis and the linear regression test for multivariate analysis.

**Results: ** The mean (SD) of REBA score was 7.7 (1.9), which means a high risk level and highlights an urgent need to change the working postures of the studied nurses. There was significant relationship between working posture and age (P = 0.003), gender (P = 0.003), regular daily exercise (P = 0.048), work experience (P = 0.003), number of shifts per month (P = 0.006) and type of operating rooms (P < 0.001) in univariate analyses. Gender and type of operating room were the predictors of working posture of nurses in multivariate analysis.

**Conclusion: ** The findings highlight the need for ergonomic interventions and educational programs to improve working posture of this study population, which can consequently lead to promotion of health and well-being of this group.

## Introduction

 In almost every workplace there are a number of job-related factors that threaten health and safety of employees. This is the case for nursing job in hospitals and clinical settings, where there are high levels of physical and mental demands in this job that threaten health status of this working group.^1,2^ Thus, in order to maintain an acceptable level of work performance and health status of employees, it is necessary to plan and organize the jobs appropriately based on scientific methods. Ergonomics is a science that can help to promote health and well-being of employees through the design of work tasks, tool and equipment and also appropriate allocation of tasks to people in their work.

 A better understanding of ergonomic risk factors in each working environment is important because such risk factors can lead to a number of adverse consequences among employees. There is evidence that inappropriate working postures can contribute to the development of musculoskeletal disorders among workers in different occupational groups.^3-5^ A number of previous studies have also shown a high prevalence of musculoskeletal symptoms in different body areas of nurses, particularly in the low back, neck, shoulders and knees.^6-12^ The high prevalence of musculoskeletal symptoms in this job may be attributed to high physical demands and inappropriate working postures that have to be maintained for a long period of time during a working shift.

 There are a number of techniques available for evaluation of physical workload of employees during their work. Postural analysis is an important technique for evaluation of work activities in this regard. However, a review of the literature indicates that the postures of operating room nurses during their work have received very limited attention, and consequently little is known about the working posture and its predictors among this working group. Research to be conducted on this issue will have important implications in terms of employees’ health and well-being and patient outcomes. The findings can also help to better understand the physical working condition of operating room nurses and identify areas that need further attention (e.g. education of employees to adopt more appropriate working postures, (re)designing of work stations, etc.). Therefore, in an attempt to address this issue, the present study was conducted to evaluate the working posture of operating room nurses and its relationship with demographic and job details of this group.

## Materials and Methods

###  Participants and Procedures

 This descriptive analytical cross-sectional study was carried out during a five month period from January 2015. The study population was consisted of operating room nurses in teaching hospitals of the Tabriz University of Medical Sciences including 2 general and 5 specialty (orthopedic, cardiac, gynecology and pediatric) centers. Being in good general health with no history of musculoskeletal injury/disease (e.g. surgery, kyphosis and scoliosis), having an associate degree or higher in nursing or operating room courses and working at least for one year in operating room were considered as inclusion criteria for the study. The study employed cluster sampling method. For this, each center was visited as a separate cluster and individual personnel numbers were recorded. Then, numbers were selected randomly by an individual who did not participate in the study. Finally, the selected numbers were matched to the numbers in the personnel list for final selection of the study participants. The number of required participants from each center was determined based on proportion to size sampling method. To determine sample size, basic information regarding working posture of nurses was obtained through a pilot study with 30 participants (r=0.318). Considering 95% confidence level, a power of 80%, and two-tailed tests, the minimum sample size was determined as 72 using G-power software. The calculated sample size was then multiplied by 2 to obtain the total sample size of 144 with respect to the design effect of 2 for cluster sampling.

###  Data collection and procedure

 Data were collected using questionnaires and direct observation of the participants during their work. The questionnaire recorded demographic details including age, gender, marital status and study major, as well as daily exercise habits of the participants. The questionnaire also covered items regarding the job including work experience, type of operating room, shift working, having a second job/responsibility, job satisfaction, and perceived pressure due to work.

 Working postures of nurses at their workstations were evaluated using the Rapid Entire Body Assessment (REBA) method,^13^ which is a reliable and validated observational method. This tool gives a specific scoring method for recording posture of each body part (e.g. neck-trunk-legs and shoulders-elbow-wrist), which is based on various static or dynamic movements, movements with rapid changes and unstable positions. The overall REBA score ranges from 1 to 15, with higher scores showing the more problematic postures. An overall REBA score relates to one of the five action levels: Action level 0 (score of 1) which means that the risk could be overlooked and there is no need to change the current status; Action level 1 (scores of 2-3) that means low risk in which change in position might be needed; Action level 2 (scores of 4-7) which means moderate risk that necessarily requires a change in position; Action level 3 (scores of 8-10) which means high risk with quick necessity to apply changes in position; and Action level 4 (scores of 11-15) which means great risk that requires urgent position change. The present study examined the working postures of operating nurses while doing three main activities in their job including retracting, transferring sets and setting up the table. The observations and recordings of working postures were carried out by two investigators, using a separate REBA assessment sheet for each operator for recording the REBA scores. The inter-rater reliability of the REBA scores was evaluated using Kappa coefficients and the results showed good reliability. This rate was 87.1 % for retracting, 89.1% for transferring the sets and 89.8% for setting up the table.

###  Data analysis

 The data were analyzed using SPSS 16.0 software (SPSS Inc., Chicago, IL, USA). The data normality was approved using Kolmogorov-Smirnov test. Descriptive statistics were presented as mean (M), standard deviation (SD), frequency (f) and percentage (%). The relationship of working postures (REBA scores) with quantitative continuous (age, work experience and number of shifts), dichotomous (gender, daily exercise and having other jobs) and multi-category (level of educational attainment and BMI) variables were assessed using *t* test, Pearson correlation coefficient and analysis of variance (ANOVA) analyses, respectively. Those variables with *P* value <0.1 were included in linear regression analysis with main effect model. Since the variables should be included quantitatively in multiple linear regression models, all of the qualitative variables were included as marker variables. *P* values <0.05 were considered as statistically significant for all statistical tests.

## Results

###  Sample characteristics

 The participants’ ages ranged from 24 to 52 years (mean=34.6 years; SD=6.6 years), and had been working in their jobs between 2 and 28 years (mean=11.2 years; SD=6.5 years). Most of the participants were women (80.3%), married (76.2%), had rotating shift work (85.7%) and were not involved in regular daily exercise (85.7%).The number of their work shifts ranged from 24 to 52 shifts per month (mean=13.7; SD=5.6).

###  Working postures

 The mean (SD) of overall REBA score for all activities evaluated in this study (retracting, transferring sets and table setup) was 7.7 (1.9), indicating that the operating room nurses were generally at high risk level. Table 1 shows the ergonomic risk levels based on REBA scores for different job activities. This table indicates that in most cases, the working posture in retracting activity was at high or very high risk levels (62.6%). This was the case for transferring sets and table setup activities, where the working postures were at high or very high risk levels in 55.9% and 48.3% of cases, respectively.

**Table 1:** Ergonomic risk levels based on REBA scores for different job activities

**Activity**	**Risk level (based on REBA scores)**				
	**Very low risk**	**Low risk**	**Moderate risk**	**High risk**	**Very high risk**
Retracting	0 (0)	1 (0.7)	54 (36.7)	76 (51.7)	16 (10.9)
Transferring sets	0 (0)	2 (1.4)	63 (42.8)	55 (37.5)	27 (18.4)
Table setup	0 (0)	3 (2.1)	73 (49.7)	59 (40.1)	12 (8.2)

Abbreviation: REBA‏; Rapid Entire Body Assessment.

Table 2 shows the relationship between study variables and working postures of the study participants. The results of the study showed significant relationship between gender and working posture (evaluated by REBA method) (*P*<0.01), so that women were more prone to awkward working postures than men. Moreover, those nurses who exercised on a regular daily basis had a better ergonomic posture than other participants (*P*<0.05). The type of operating room had also a significant effect on body posture of the study participants (*P*<0.001). This finding indicated that those nurses who worked in cardiac operating room had higher REBA scores than other participants. The results also showed a positive relationship between age (*P*<0.01) and work experience (*P*<0.01) with working posture of the studied participants. Finally, there was a negative relationship between the number of work shifts and working posture of nurses (*P*<0.001).

**Table 2:** Relationship between study variables and REBA scores

**Variables**	**Number**	**%**	**REBA score (M±SD)**	***P *** **value**
Gender				
Male	29	19.7	6.82±2.15	0.003
Female	118	80.3	8.01±1.85	
Education level				
Associate	59	59	7.62±2.02	0.733
BSc	86	58.5	7.87±1.93	
MSc or higher	2	1.4	8±2.35	
Major				
Operating room	86	58.5	7.68±1.86	0.486
Nurse	61	41.5	7.91±2.10	
Type of operating room				
Orthopedic	33	22.4	7.33±2.08	<0.001
General	51	34.7	6.64±1.36	
Skin and burns	4	2.7	6.66±0.98	
Cardiac	20	13.6	10.26±1.05	
Pediatric	12	8.2	7.66±0.91	
Gynecology	27	18.4	8.82±1.62	
Work shifts				
Morning	19	12.9	7.91±1.77	0.767
Evening	2	1.4	8.66±3.29	
Rotating	126	85.7	7.74±1.98	
Daily exercise				
Yes	21	14.3	7.14±1.83	0.048
No	126	85.7	7.88±1.97	
Marital status				
Single	35	23.8	7.42±2.03	0.629
Married	112	76.2	7.88±1.94	
Perceived work pressure				
Yes	128	87.1	7.74±1.91	0.629
No	19	12.9	7.98±2.31	
Second job/responsibility				
Yes	48	32.7	7.59±2.15	0.334
No	99	67.3	7.86±1.87	
Job satisfaction				
Low	21	14.3	7.28±1.75	0.451
Medium	104	7.70	7.88±2.07	
High	22	15.0	7.75±1.60	
**Quantitative variables**	**M** **±** **SD**		**Correlation coefficient**	***P*** ** value**
Age(years)	34.65±6.61		0.245	0.003
Work experience (years)	11.26±6.50		0.243	0.003
Number of shifts	31.73±5.64		-0.224	0.006

‏ Abbreviation: REBA‏; Rapid Entire Body Assessment.

 Those significant variables in univariate analysis (with *P*<0.1) were also included in linear regression model to determine the predictors of working postures of operating room nurses under study (Table 3). Among the included variables, gender and type of operating room were found to be the predictors of working posture among the study population. The findings indicated that males had 1.012 higher scores than females, which means better working postures in this regard. Considering the fact that the posture risk level of those employees working at cardiac operating room was higher than others, this was considered as a reference. Thus, the personnel of orthopedic, general, skin and burns and pediatric operating rooms had 1.228, 2.014, 2.173 and 1.069 higher scores than those working at cardiac operating room. Based on the results, the predictive factors could predict 43.5% of variance changes in working postures.

**Table 3:** Estimates of linear regression for predictive factors of working posture

**Variables**	**Regression coefficient**	**95% CI**		***P*** ** value**
		**Lower bound**	**Upper bound**	
Gender				0.006
Male	-1.012	-1.728	-0.295	0.006
Female	Reference			
Type of operating room				<0.001
Orthopedic	-1.228	-2.051	-0.405	0.004
General	-2.014	-2.792	-1.236	0.0001
Skin and burns	-2.173	-3.876	-0.470	0.013
Gynecology	1.444	0.498	2.389	0.003
Pediatric	-1.069	-2.143	0.006	0.051
Cardiac	Reference			
Daily exercise				0.858
Yes	-0.071	-0.852	0.711	0.858
No	Reference			
Age	0.013	-0.084	0.110	0.790
Work experience	0.007	-0.091	0.100	0.890
Number of shifts	0.023	-0.029	0.079	0.382

## Discussion

 The present study was carried out to evaluate the working posture and its predictors among operating room nurses in Iran, Tabriz. The main findings of the study were that the working posture of the studied population was not ergonomically appropriate and there was significant relationship between working posture and age, gender, regular daily exercise, number of shifts per month and type of operating room in univariate analyses. Gender and type of operating room were the most important predictors of working posture of nurses in multivariate linear regression analysis.

 One of the interesting findings of the present study was that the overall REBA score among the studied nurses was 7.7 which is relatively high and indicates abnormal working posture among this working group. This finding highlight that in most cases nurses were at high risk level and needed urgent and prompt change in their working posture. These findings clearly indicate that the operating room nurses are exposed to a high level of physical ergonomic risk factors in their working environment that need to be considered when evaluating their working work. Among the different job activities evaluated in this study, working posture of the majority of employees during the retracting activity (62.6%) was found to be more stressful than other job activities. However, other job activities including transferring sets and table setup activities were also found to be not ergonomically appropriate and were classified as high risk level for approximately half of the participants (e.g. 55.9% and 48.3% for transferring sets and table setup activities, respectively). These findings indicate that how challenging is the working posture of the operating room nurses, and therefore ergonomic interventions are needed to improve the working condition of this group.

 Several previous studies have shown that inappropriate working posture can lead to the development of musculoskeletal symptoms in different occupational groups.^3-5^ The results of the present study also indicated that, in most cases, the working posture of the operating room nurses was not appropriate. This finding may suggest that preventive measures for reducing musculoskeletal complaints in different body parts of the operating room nurses should be aimed at improving the working posture and working conditions of this occupational group.

 As shown in this study, there was also a positive relationship between age, regular daily exercise and work experience of the studied nurses and their working posture in univariate analyses. It was shown in this study that those participants with higher age and work adopt more awkward working postures during their work. This finding provide further evidence to the need for more flexible solutions to decrease challenges associated with physically demanding working conditions for the older employees.^14^ The results of a study conducted among nurses in Swedish hospitals indicated that younger people could adapt themselves to safety techniques easier and faster than middle-aged people.^15^ The authors argued that as the person’s age goes up, joint movement and physical capacity tends to decrease and it can weaken the work techniques used by that person, and this can consequently lead to the adoption of more awkward working postures and to increased prevalence of musculoskeletal symptoms.^15^ It is also possible that, compared to nurses with less work experience, those with more years of experience are less familiar with ergonomic principles and proper methods of their task performance. This is of upmost importance and highlights the need for education of those employees who are less aware about the consequences of adopting inappropriate and awkward postures during their work as this can lead to several health problems to them. The finding that those nurses who exercised on a regular daily basis adopted more ergonomically working postures may also highlight the role of exercise in preventing awkward postures. Taken together, these findings add to the understanding of the factors that influence working postures of operating room nurses and have important implications for designing ergonomic interventions and measures for health promotion of this working group.

 The results of multivariate linear regression analysis indicted that among the studied variables, gender and type of operating room were the two important predictors of the adopted postures by the operating room nurses during their work. The findings indicated that females were more likely to adopt awkward postures during their work than their male counterparts. This finding highlights the educational needs of employees and suggests that female employees may be at greater need for ergonomic interventions and educational programs regarding the correct methods of their task performance. The results also indicated that those nurses working at cardiac ward were more likely to adopt inappropriate working postures than those working at other areas. This may be attributed to the type of job activities performed in this ward which involves long-duration operations (e.g. heart surgeries) as well as moving large and heavy sets in these operating rooms that consequently can lead to sustained static loading and postural stress in this group.

 The present study was conducted among operating room nurses, and therefore generalizing of the findings to the nurses in other units should be done cautiously. Further studies on this issue among nurses in other units and settings seem to be required. Moreover, the predicting variables in this study were limited to demographic and contextual features. It would seem also advisable in future studies to evaluate other possible variables as well as environmental factors that may influence working postures of this working population.

## Conclusion

 The relatively high REBA scores in operating room nurses in this highlight a poor working condition and suggest that the nurses’ postures at their work stations need urgent investigation and prompt changes are required. A number of significant relationships between working postures and demographic and job characteristics in this study add to the understanding of the working posture of operating room nurses and emphasize the need for ergonomic interventions and educational programs for improving the health and well-being of this working group.

## Ethical approval

 Permission for this study was obtained from the hospital authorities involved and the study protocol was approved by the ethics committee in the Tabriz University of Medical Sciences. Each participant was informed about the aims of the study and signed a consent form before participation and their data were kept confidential. Participation in the study was voluntary and the participants were free to leave the study at any stage.

## Competing interests

 The authors declare that there is no conflict of interests.

## Acknowledgments

 This study was funded by the Tabriz University of Medical Sciences (Grant No. 5/4/11718). The authors also acknowledge the kind collaboration of operating room nurses of the Tabriz teaching hospitals who enthusiastically participated in this study.
